# Particle Size Measurement Using Dynamic Light Scattering at Ultra-Low Concentration Accounting for Particle Number Fluctuations

**DOI:** 10.3390/ma14195683

**Published:** 2021-09-29

**Authors:** Mengjie Wang, Jin Shen, John C. Thomas, Tongtong Mu, Wei Liu, Yajing Wang, Jinfeng Pan, Qin Wang, Kaishi Liu

**Affiliations:** 1 School of Electrical and Electronic Engineering, Shandong University of Technology, Zibo 255049, China; wangmengjie97@hotmail.com (M.W.); john@group-scientific.com.au (J.C.T.); tongtongslxm@126.com (T.M.); weikey@sdut.edu.cn (W.L.); wangyajing@sdut.edu.cn (Y.W.); pjfxx@sdut.edu.cn (J.P.); wangqin05192021@163.com (Q.W.); lleo66@163.com (K.L.); 2 Group Scientific Pty Ltd., 23 Pine Lodge Crescent, Grange, SA 5022, Australia

**Keywords:** dynamic light scattering, ultra-low particle concentration, kernel function reconstruction, baseline reset

## Abstract

Dynamic light scattering (DLS) is a popular method of particle size measurement, but at ultra-low particle concentrations, the occurrence of number concentration fluctuations limits the use of the technique. Number fluctuations add a non-Gaussian term to the scattered light intensity autocorrelation function (ACF). This leads to an inaccurate particle size distribution (PSD) being recovered if the normal DLS analysis model is used. We propose two methods for inverting the DLS data and recovering the PSDs when number fluctuations are apparent. One is to directly establish the relationship between the non-Gaussian ACF and the PSD by the kernel function reconstruction (KFR) method while including the non-Gaussian term to recover the PSD. The other is to remove the effect of the non-Gaussian term in the ACF by the baseline reset (BR) method. By including the number fluctuation term, the ideal recovered PSD can be obtained from the simulated data, but this will not happen in the experimental measurement data. This is because the measured intensity ACF contains more noise than the simulated ACF at ultra-low concentration. In particular, the baseline noise at the tail of long delay time of ACF overwhelms the number fluctuation term, making it difficult to recover reliable PSD data. Resetting the baseline can effectively remove the digital fluctuation term in ACF, which is also a feasible method to improve PSD recovery under ultra-low concentration. However, increasing noise at ultra-low concentrations can lead to errors in determining an effective baseline. This greatly reduces the accuracy of inversion results. Results from simulated and measured ACF data show that, for both methods, noise on the ACF limits reliable PSD recovery.

## 1. Introduction

Dynamic light scattering (DLS) was pioneered in the 1960s [1,2,3] and is now also commonly called photon correlation spectroscopy (PCS) because the experiment is done using digital correlation of photon count signals [4,5]. This technique has been widely applied to measure particle size in many fields such as materials, chemical industry, food, biology and medicine [6,7,8,9,10]. It is an effective technology [11] and a standard method for measuring the particle size and distribution of submicron particles in suspension [12,13,14,15]. Particle size and particle size distribution (PSD) determination essentially occurs by measuring the diffusion coefficients of the particles undergoing Brownian motion in a small scattering volume. This is done by analyzing the timescale of the fluctuations in the intensity of the light scattered from the particles. The particle concentration is high enough that the number of particles in the scattering volume is essentially constant. For an ultra-low concentration particle system, there are less than ~50 diffusively moving particles in the scattering volume, and particle diffusion in and out of the volume may result in substantial changes in the relative local particle concentration, which will also give rise to changes in the scattered light intensity. Thus, intensity fluctuations will arise now from both the Brownian motion of the particles and the fluctuations in the number concentration of particles [16,17,18,19]. These fluctuation components will occur on different timescales and with different amplitudes. Willemse et al. proposed a method where the output signals of the photon detectors are divided by high and low frequencies by using the frequency difference between signals caused by Brownian motion and fluctuation of particle number. They removed the number fluctuation noise located in the low frequency band from signals and carried out a series of studies [20,21,22,23,24]. Although this method is feasible in theory, additional devices are needed between detector and correlator in the DLS measurement system and additional noise will be introduced. Furthermore, it changes the real-time signal acquisition and measurement nature of DLS as the signal represented by the pulse density is low-pass filtered before entering the photon correlator, which makes DLS measurement at low concentration different to what is normally done. This method is difficult to implement and operate in practice and has not been realized in a commercial particle size analyzer.

The ideal DLS measurement at ultra-low concentration is to obtain the PSD information by analyzing the light intensity autocorrelation function (ACF) data calculated in real-time by the correlator without changing the real-time operation mechanism of DLS. For ultra-low concentration samples, the amplitude change of the scattered field is no longer a Gaussian random process due to the small number of particles in the scattering volume [25]. In this case, the light intensity ACF and electric field ACF are no longer related by the Siegert relation. However, an additional relaxation term can be added to the relation to account for number fluctuations. If provision is not made for this term, the PSD results will be meaningless. For this reason, it is clearly stipulated in the corresponding international standards [14] that the number of particles in the measured volume must be kept constant. If there are large particles or the sample concentration is too low in the scattering volume, this situation may contribute an artifact to the PSD.

Recently, interest in the measurement of ultra-low concentration samples has increased [26,27,28]. Earlier studies suggest that there are two approaches to obtain PSD information of ultra-low concentration samples. One is to construct an equivalent electric field ACF model using the modified Siegert relation, and reconstruct the kernel function of the inversion equation, and then obtain the PSD. The second is to effectively separate the contribution of particle number fluctuation in the measured intensity ACF, and then recover the PSD through the usual data processing method. In this paper we investigate the two ways to carry out DLS measurements at ultra-low concentrations through theoretical analysis, numerical simulation and experimental verification. In addition, for DLS measurement at ultra-low concentration, the key inhibiting factors were analyzed and feasible ways to improve the accuracy were discussed.

## 2. Theory and Methods

### 2.1. Classical DLS Theory and Number Fluctuations in the Scattering Volume

Light scattered from a suspension of Brownian particles exhibits intensity fluctuations [15]. The time scale of the fluctuations is related to the diffusion coefficient of the Brownian particles, which is related to the size of the particles. These fluctuations can be characterized by the light intensity ACF [15]

(1) $$G^{\left(2\right)}\left(\tau \right)=\langle i\left(t\right)\cdot i\left(t+\tau \right)\rangle =\underset{T_{0}\to \infty }{\lim}\int_{0}^{T_{0}}i\left(t\right)\cdot i\left(t+\tau \right)dt/T_{0}.$$

Here, i(t) is the scattered light intensity, $T_{0}$ and τ are measurement time and delay time, respectively. The intensity ACF can be obtained by photon correlation. For a Gaussian light field, the normalized light intensity ACF, $g^{\left(2\right)}\left(\tau \right)$, and the normalized electric field ACF, $g^{\left(1\right)}\left(\tau \right)$, satisfy the Siegert relation

(2) $$g^{\left(2\right)}\left(\tau \right)=1+\beta {\left|g^{\left(1\right)}\left(\tau \right)\right|}^{2},$$

where β is an instrumental coherence factor. For Brownian particles and, in the absence of number fluctuations, the electric field ACF can be expressed as

(3) $$g^{\left(1\right)}\left(\tau \right)=\exp\left(-\Gamma \tau \right).$$

Here, $\Gamma =D_{T}q^{2}$ is the decay constant. $D_{T}$ is the diffusion coefficient and is related to particle diameter d and medium viscosity η by the Stokes–Einstein relation $D_{T}=\frac{k_{B}T}{3\pi \eta d}$. Here, $k_{B}$ is the Boltzmann constant, and T is the absolute temperature of the scattering medium. q is the scattering vector, whose amplitude is $|q|=\frac{4\pi n}{\lambda_{0}}\sin\frac{\theta }{2}$. $\lambda_{0}$, n, and θ are wavelength of incident light in vacuum, refractive index, and scattering angle of solution, respectively.

For polydisperse particle systems, the relation between particle size and the normalized electric field ACF is [15,29]

(4) $$g^{\left(1\right)}\left(\tau \right)=\int_{0}^{\infty }G\left(\Gamma \right)\exp\left(-\Gamma \tau \right)d\Gamma ,$$

where G(Γ) is normalized intensity distribution function of Γ. Using the Stokes–Einstein relation and substituting in the various parameters allows Equation (4) to be written in discrete form as,

(5) $$g^{\left(1\right)}\left(\tau_{j}\right)=\sum_{i=1}^{M}\exp\left(-\frac{16\pi n^{2}k_{B}T}{3\eta \lambda_{0}{}^{2}d_{i}}\sin^{2}\left(\frac{\theta }{2}\right)\tau_{j}\right)f\left(d_{i}\right).$$

Here, $g^{\left(1\right)}\left(\tau_{j}\right)$ is the normalized electric field ACF, j(1≤j≤Ch) is the correlator channel number. $f\left(d_{i}\right)$ is the discrete PSD and satisfies $\sum_{i=1}^{M}f\left(d_{i}\right)=1$. $d_{i}\left(i=1,2,\ldots ,M\right)$ is the particle diameter. Equation (5) can be simplified as

(6) g=Af.

Here, g is the vector composed of the normalized electric field ACF data, whose elements are $g^{\left(1\right)}\left(\tau_{j}\right)$ and dimension is Ch×1. The elements of f, a vector made up of discrete PSDs, are $f\left(d_{i}\right)$. The dimension of f is M×1 and A is the kernel matrix corresponding to the electric field ACF data, whose dimension is Ch×M. The elements of A are $A\left(j,i\right)=\exp\left(-\frac{16\pi n^{2}k_{B}T}{3\eta \lambda_{0}{}^{2}d_{i}}\sin^{2}\left(\frac{\theta }{2}\right)\tau_{j}\right)$. Equation (6) is a Fredholm integral equation of the first kind, and is a typical ill-posed problem that can be solved by the Tikhonov regularization method. The ill-posed problem is transformed into a well-posed problem, and the approximate solution closest to the real solution of Equation (6) is obtained by optimizing the objective function,

(7) $$M_{\alpha }\left(f,g\right)={\left\|Af\;-\;g\right\|}_{2}^{2}+\alpha {\left\|Lf\right\|}_{2}^{2},s.t.f\geq 0.$$

Here, α, L, ${\left\|\cdot \right\|}_{2}^{2}$, and ${\left\|Lf\right\|}_{2}^{2}$ are regularization parameter, regularization matrix, Euclidean norm, and penalty factor, respectively.

At ultra-low concentrations, when number fluctuations are significant, the scattered field amplitude is no longer Gaussian, and the intensity ACF and field ACF no longer satisfy the usual Siegert relation. Under this condition, the relation between the intensity ACF and the field ACF can be expressed as [30]

(8) $$g_{d}^{\left(2\right)}\left(\tau \right)=1+\beta {\left|g^{\left(1\right)}\left(\tau \right)\right|}^{2}+\frac{\gamma }{\langle N\rangle }{\left(1+\frac{4D_{T}\tau }{\omega_{0}{}^{2}}\right)}^{-1}{\left(1+\frac{4D_{T}\tau }{a^{2}}\right)}^{-\frac{1}{2}},$$

where $\gamma =2^{-3/2}$ is a constant related to the effective scattering volume [30]. 〈N〉 is the average number of particles in the scattering volume, $\omega_{0}$ is the waist radius of the laser beam, and a is the radius of the detector aperture. In Equation (8), the second term on the right represents the contribution of Brownian motion to the ACF, and the third term is the number fluctuation term. The latter is usually referred to as the non-Gaussian term. For 〈N〉≥50 particles in the scattering volume, the term is negligible and can be ignored for routine DLS measurements. Comparing Equations (8) and (2), it can be seen that the number fluctuation term also contains PSD information and that this term becomes larger as the number concentration decreases making it impossible to obtain the field ACF using the usual Siegert relation at very low particle concentrations. Also, the wrong PSD will be recovered by using the kernel function matrix in Equation (6). Thus, the analysis of DLS is not straight forward at ultra-low concentration.

### 2.2. PSD Recovery from DLS Measurements at Ultra-Low Concentrations

#### 2.2.1. Kernel Function Reconstruction of Inversion Equation

Equation (8) shows that both Brownian motion and particle number fluctuations potentially provide particle size information. If we can construct the field ACF to include Brownian motion and number fluctuations and reconstruct the corresponding kernel function matrix on the basis of Equation (8), we establish the inversion equation relationship between the equivalent field ACF and the PSD. The PSD information would be obtained from both Brownian motion and number fluctuations.

In discrete form, Equation (8) can be written as

(9) $$g_{d}^{\left(2\right)}\left(\tau_{j}\right)=1+\beta \exp\left(-\frac{32\pi n^{2}k_{B}T}{3\eta \lambda_{0}{}^{2}d}\sin^{2}\left(\frac{\theta }{2}\right)\tau_{j}\right)+\frac{\gamma }{\langle N\rangle }{\left(1+\frac{{4k}_{B}T\tau_{j}}{3\pi \eta d\omega_{0}{}^{2}}\right)}^{-1}{\left(1+\frac{4k_{B}T\tau_{j}}{3\pi \eta da^{2}}\right)}^{-\frac{1}{2}}.$$

For a polydisperse particle system, the intensity ACF can be expressed as

(10) $$g_{d}^{\left(2\right)}\left(\tau_{j},d_{i}\right)=1+\beta \sum_{i=1}^{M}\left(\exp(-\frac{32\pi n^{2}k_{B}T}{3\eta \lambda_{0}{}^{2}d_{i}}\sin^{2}\left(\frac{\theta }{2}\right)\tau_{j})+\frac{\gamma }{\beta \langle N\rangle }{\left(1+\frac{{4k}_{B}T\tau_{j}}{3\pi \eta d_{i}\omega_{0}{}^{2}}\right)}^{-1}{\left(1+\frac{4k_{B}T\tau_{j}}{3\pi \eta d_{i}a^{2}}\right)}^{-\frac{1}{2}}\right)f\left(d_{i}\right).$$

If we define

(11) $$A_{1}\left(\tau_{j},d_{i}\right)=\exp\left(-\frac{32\pi n^{2}k_{B}T}{3\eta \lambda_{0}{}^{2}d_{i}}\sin^{2}\left(\frac{\theta }{2}\right)\tau_{j}\right),$$

(12) $$e\left(\tau_{j},d_{i}\right)=\frac{\gamma }{\beta \langle N\rangle }{\left(1+\frac{{4k}_{B}T\tau_{j}}{3\pi \eta d_{i}\omega_{0}{}^{2}}\right)}^{-1}{\left(1+\frac{4k_{B}T\tau_{j}}{3\pi \eta d_{i}a^{2}}\right)}^{-\frac{1}{2}},$$

and by analogy with the Siegert equation we can write an equivalent field ACF as

(13) $$g_{1}\left(\tau_{j}\right)=\sum_{i=1}^{M}\sqrt{A_{1}\left(\tau_{j},d_{i}\right)+e\left(\tau_{j},d_{i}\right)}f\left(d_{i}\right),$$

The inversion equation in the form of Equation (6) is obtained, and the reconstructed kernel function matrix is expressed as

(14) $$A_{r}=\sqrt{A_{1}+e},$$

and its elements are

(15) $$A_{r}\left(j,i\right)=\sqrt{\exp\left(-\frac{32\pi n^{2}k_{B}T}{3\eta \lambda_{0}{}^{2}d_{i}}\sin^{2}\left(\frac{\theta }{2}\right)\tau_{j}\right)+\frac{\gamma }{\beta \langle N\rangle }{\left(1+\frac{{4k}_{B}T\tau_{j}}{3\pi \eta d_{i}\omega_{0}{}^{2}}\right)}^{-1}{\left(1+\frac{4k_{B}T\tau_{j}}{3\pi \eta d_{i}a^{2}}\right)}^{-\frac{1}{2}}}.$$

The PSD can be obtained from Equation (7).

#### 2.2.2. Intensity ACF Baseline Reset

Comparing Equation (2) with Equation (8), it can be seen that the last term on the right of Equation (8), the number fluctuation term, is not present for DLS measurements at higher concentrations. The number fluctuation term has an amplitude that decreases with concentration and a long decay time.

We define the number fluctuation term in the intensity ACF as

(16) $$g^{\prime }\left(\tau \right)=\frac{\gamma }{\langle N\rangle }{\left(1+\frac{4D_{T}\tau }{\omega_{0}{}^{2}}\right)}^{-1}{\left(1+\frac{4D_{T}\tau }{a^{2}}\right)}^{-\frac{1}{2}}.$$

**Figure 1:**
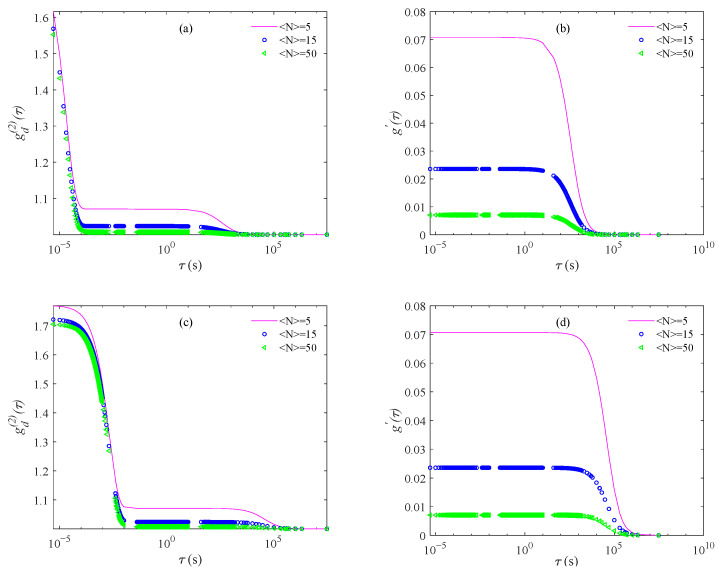
Theoretical (**a**) intensity ACFs $g_{d}^{\left(2\right)}\left(\tau \right)$ and (**b**) number fluctuation terms $g^{\prime }\left(\tau \right)$ for 10 nm particles, (**c**) intensity ACFs $g_{d}^{\left(2\right)}\left(\tau \right)$ and (**d**) number fluctuation terms $g^{\prime }\left(\tau \right)$ for 1000 nm particles at average particle number 〈N〉 = 5, 15, and 50 in the scattering volume.

Figure 1 shows the theoretical intensity ACFs, $g_{d}^{\left(2\right)}\left(\tau \right)$, and number fluctuation terms, $g^{\prime }\left(\tau \right)$, for 10 nm and 1000 nm particles at average particle number 〈N〉 = 5, 15, and 50 in the scattering volume.

Figure 1 shows that the number fluctuation and Brownian motion terms contribute significantly different attenuation processes to the intensity ACF. This can be further seen in Table 1, which shows the delay time, $t_{d}$, for the number fluctuation term to decrease by 10^−3^ and its decrease in $t_{c}$, the Brownian motion decay time. It is observed that the largest magnitude variation in $g^{\prime }\left(\tau \right)$ over the time $t_{c}$, is only on the order of 10^−8^ for both the 10 and 1000 nm particles when 〈N〉=5. In other words, on the timescale $t_{c}$, number fluctuations essentially contribute a small constant term to the ACF. This suggests that we could obtain a PSD by removing the number fluctuation term by resetting the baseline of $g_{d}^{\left(2\right)}\left(\tau \right)$ from 1.0 and then calculate $g^{\left(1\right)}\left(\tau \right)$ using the usual Siegert relation.

**Figure 2:**
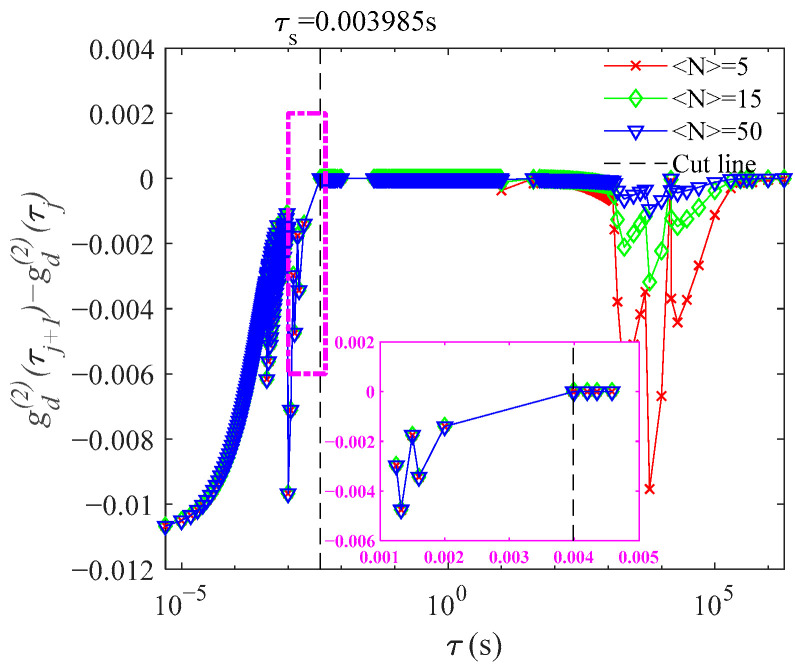
The truncation point $\tau_{s}$ determined by the difference between $g_{d}^{\left(2\right)}\left(\tau_{j+1}\right)$ and $g_{d}^{\left(2\right)}\left(\tau_{j}\right)$.

**Figure 3:**
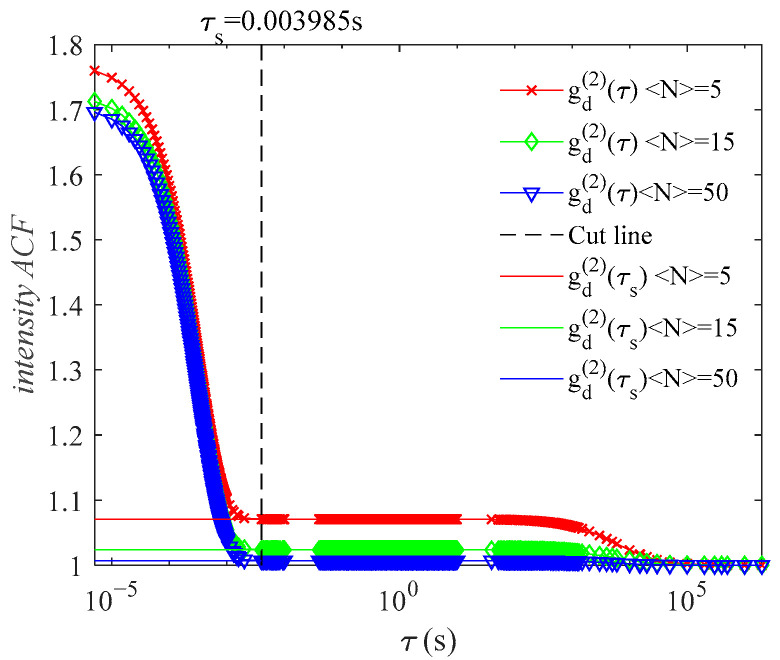
The new baseline $g_{d}^{\left(2\right)}\left(\tau_{s}\right)$ of $g_{d}^{\left(2\right)}\left(\tau \right)$ determined by $\tau_{s}$.

To determine a new, adjusted baseline, we can examine the change in adjacent channels of $g_{d}^{\left(2\right)}\left(\tau \right)$ and find the truncation point where $g^{\left(2\right)}\left(\tau \right)$ at $\tau \geq t_{c}$. Figure 2 shows the difference between adjacent channels, $g_{d}^{\left(2\right)}\left(\tau_{j+1}\right)-g_{d}^{\left(2\right)}\left(\tau_{j}\right)$, with the delay time of $g^{\left(2\right)}\left(\tau \right)$. At $\tau_{j}=\tau_{s}$, when $g_{d}^{\left(2\right)}\left(\tau_{j+1}\right)-g_{d}^{\left(2\right)}\left(\tau_{j}\right)\leq 10^{-3}$, $g_{d}^{\left(2\right)}\left(\tau \right)$ has decayed to $g^{\prime }\left(\tau \right)$, the effective baseline term due to number fluctuations. The new ACFs, $g^{\left(2\right)}\left(\tau \right)$, with new baseline $g_{d}^{\left(2\right)}\left(\tau_{s}\right)$ are obtained as shown in Figure 3. Then, $g^{\left(1\right)}\left(\tau \right)$ is calculated by the usual Siegert relation and the PSD is obtained by Equation (7). In the actual DLS measurement, four consecutive delay channels are required to satisfy $g_{d}^{\left(2\right)}\left(\tau_{j+1}\right)-g_{d}^{\left(2\right)}\left(\tau_{j}\right)\leq 10^{-3}$ in order to minimize the effect of noise on the ACF in choosing the correct baseline point.

## 3. Results

### 3.1. Simulation

The usual method, the kernel function reconstruction (KFR) method, and the baseline reset (BR) method were compared using eight samples of simulated data consisting of two unimodal PSDs at ultra-low concentration. To simulate real conditions, noise at levels of 0, 10^−4^, 10^−3^, 10^−2^, and 10^−1^ was added to the ACF data. In analyzing the ACF data to reconstruct the PSDs, both the usual and the BR methods used the $A_{1}$ matrix (Equation (11)) and the KFR method used the $A_{r}$ matrix (Equation (15)).

Johnson’s *S_B_* function [31] was used to simulate PSDs as follows:

(17) $$f\left(d\right)=\frac{\sigma }{\left(d_{\max}-d_{\min}\right)\sqrt{2\pi }}{\left[t\left(1-t\right)\right]}^{-1}\exp\left[-0.5{\left[\mu +\sigma \ln\left(\frac{t}{1-t}\right)\right]}^{2}\right].$$

Here, *t* is the normalized particle size satisfying $t=\frac{d-d_{\min}}{d_{\max}-d_{\min}}$, where $d_{\max}$ and $d_{\min}$ denote maximum and minimum particle size, respectively. σ and μ are distribution parameters.

The intensity ACF was obtained using Equation (10). Gaussian random noise was

(18) $$G_{noise}^{\left(2\right)}\left(\tau \right)=G^{\left(2\right)}\left(\tau \right)+\delta n\left(\tau \right).$$

Here, $G_{noise}^{\left(2\right)}\left(\tau \right)$ is the noisy intensity ACF, *δ* is the noise standard deviation, and n(τ) denotes Gaussian random noise.

The ACF data were simulated with *λ*_0_ = 532 nm, *ω*_0_ = 54 μm, *a* = 200 μm, *T* = 298.15 K, *n* = 1.334, *θ* = 90°, k_B_ = 1.3807 × 10^−23^ J/K, *η* = 0.89 × 10^−3^ cP, and *β* = 0.9. A discrete PSD with *M* = 150 data points was used. Four concentration values were used, 〈N〉 = 6, 12, 24, and 48.

Table 2 summarizes the properties of the simulated PSDs. *P* is the peak position of the PSD. To characterize the accuracy of PSD recovery, we introduce two indices: the relative error of peak position (*E_P_*) and the PSD recovery error (*V_E_*). These two indices are defined as follows:

(19) $$E_{P}=\frac{\left|P_{true}-P_{meas}\right|}{P_{true}},$$

(20) $$V_{E}={\left(\frac{\sum_{1}^{M}{\left[f_{true}\left(d_{i}\right)-f_{meas}\left(d_{i}\right)\right]}^{2}}{M}\right)}^{1/2},$$

where $f_{true}\left(d_{i}\right)$ is the true PSD; $f_{meas}\left(d_{i}\right)$ is the recovered PSD.

**Figure 4:**
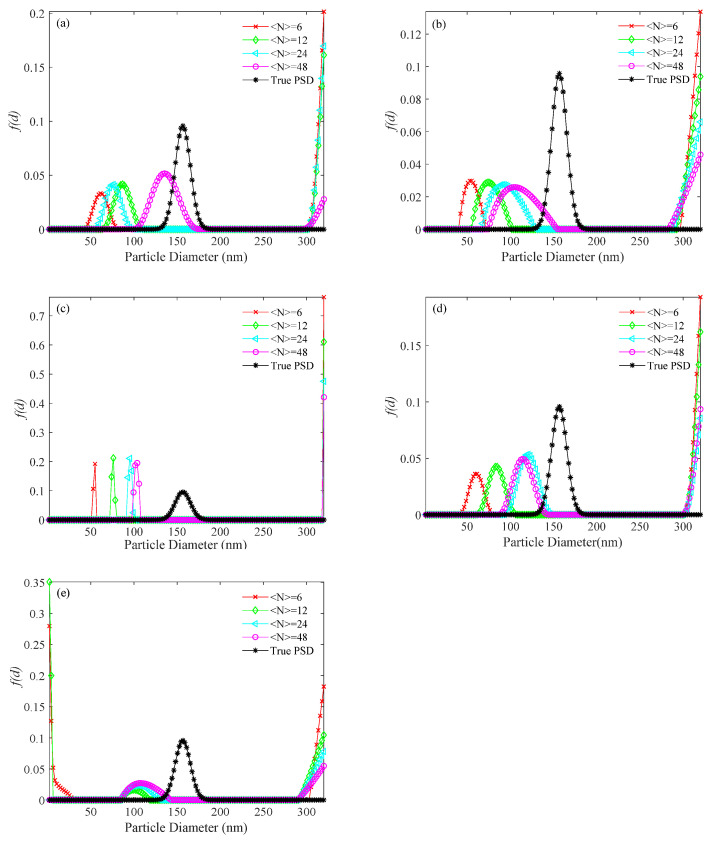
PSD recovered by the usual method for a 156 nm unimodal sample at number concentrations of <*N*> = 6, 12, 24, 48, and different levels of noise. (**a**) 0, (**b**) 10^−4^, (**c**) 10^−3^, (**d**) 10^−2^, and (**e**) 10^−1^ noise levels.

**Figure 5:**
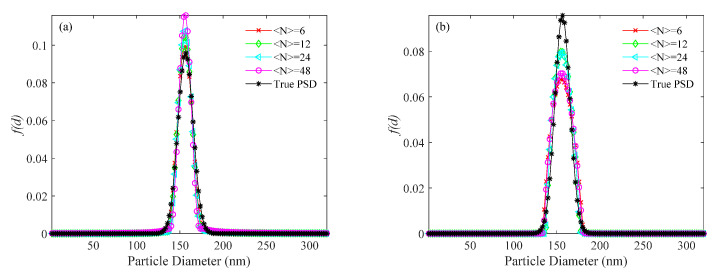

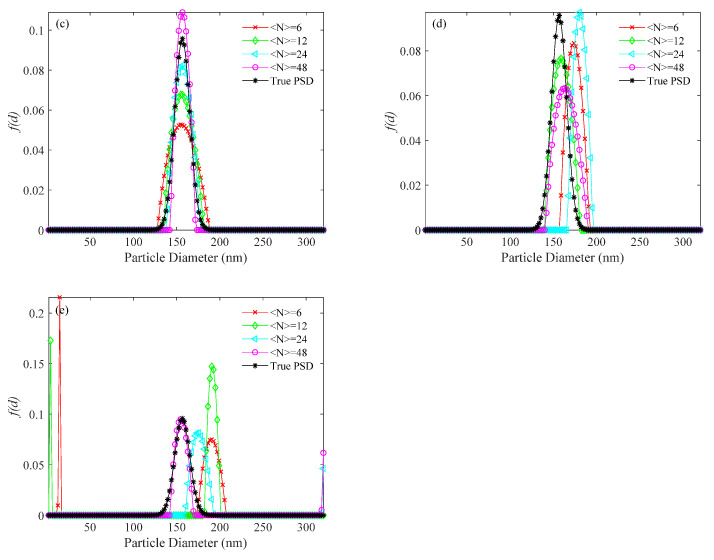
PSD recovered by the KFR method for a 156 nm unimodal sample at number concentrations of <*N*> = 6, 12, 24, 48, and different levels of noise. (**a**) 0, (**b**) 10^−4^, (**c**) 10^−3^, (**d**) 10^−2^, and (**e**) 10^−1^ noise levels.

**Figure 6:**
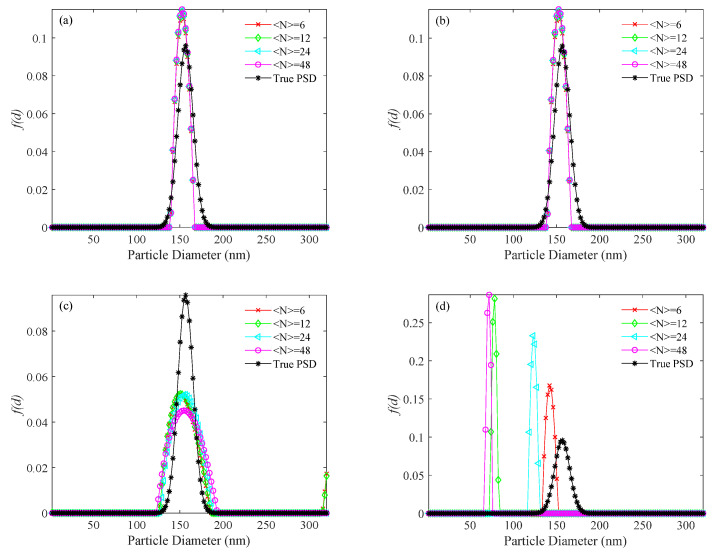

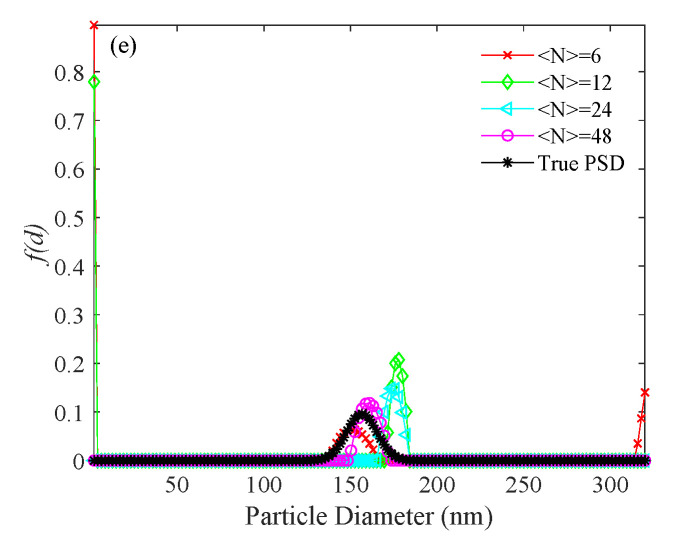
PSD recovered by the BR method for a 156 nm unimodal sample at number concentrations of <*N*> = 6, 12, 24, 48, and different levels of noise. (**a**) 0, (**b**) 10^−4^, (**c**) 10^−3^, (**d**) 10^−2^, and (**e**) 10^−1^ noise levels.

For the simulated 156 nm PSD, the PSDs recovered by the usual method, the KFR method and the BR method are shown in Figure 4, Figure 5 and Figure 6, respectively. Table 3 shows the performance parameter values of the true and recovered PSDs. *P*_U_, *E_P_*_U_, and *V_E_*_U_ are the parameters of PSD obtained by the usual method; *P*_KFR_, *E_P_*_KFR_, and *V_EKFR_* are the parameters of PSD obtained by the KFR method; *P*_BR_, *E_P_*_BR_, and *V_E_*_BR_ are the parameters of PSD obtained by the BR method. Figure 4 shows that the PSDs obtained by the usual method have false peaks much larger than the real peaks for all values of 〈N〉 investigated. The significant deviation of the main peak from the actual position is caused by the large false peak. Also, with increasing noise level, the PSDs obtained by the usual method deviate more from the true PSDs. In Figure 5, an ideal inversion result is obtained by the KFR method at zero noise level. It can be seen from Table 3 that the relative error (*E_P_*_KFR_) and the recovery error (*V_E_*_KFR_) are only 0.01 and 0.002, even when the concentration is as low as 〈N〉=6. At concentration 〈N〉=24, the KFR method gives a close estimation to the true PSD. Figure 5 and Table 3 also show that the inversion results of the KFR method are not significantly affected by noise. At low noise level (10^−3^), even if the sample concentration is extremely low (〈N〉=6), *E_P_*_KFR_ increases considerably less than *E_P_*_U_ and *V_E_*_KFR_ is limited to <0.01. As the noise level increases to 10^−2^, the *E_P_*_KFR_ and *V_E_*_KFR_ show a clear increase. In Table 3, the performance parameter values of the recovered PSDs with the BR method are clearly worse than with the KFR method at the same sample concentrations and noise levels. When the noise level is 0, the relative error (*E_P_*_BR_) of the BR method is twice that of the KFR method and the recovery error (*V_E_*_BR_) can be up to four times that of the KFR method at very low concentrations. Moreover, with increasing noise, the differences of *E_P_* and *V_E_* between the two methods tends to increase. It can also be seen that, with increasing noise, the BR method moves the peak to smaller sizes and broadens the PSD.

**Figure 7:**
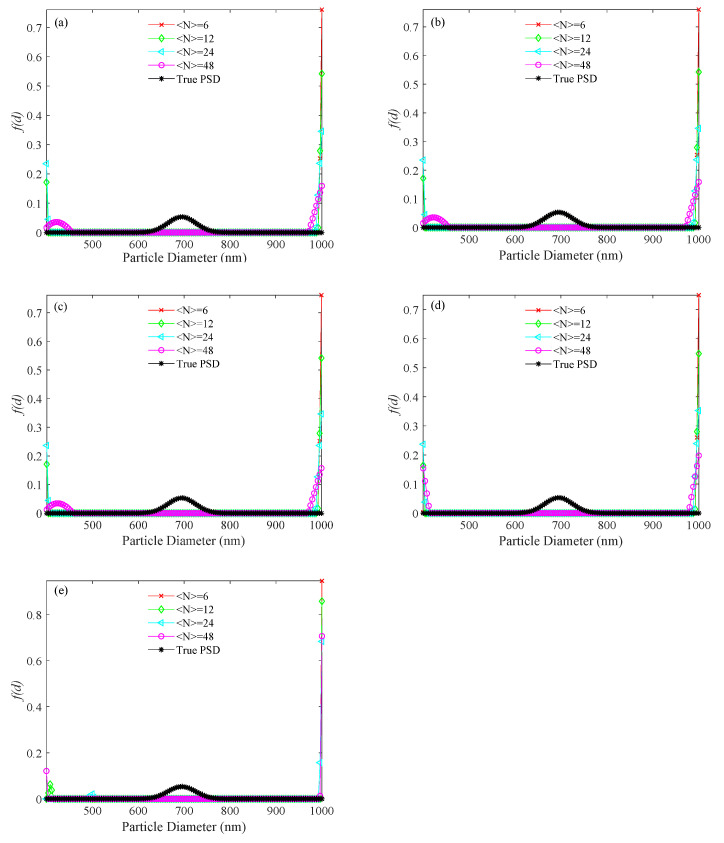
PSD recovered by the usual method for a 696 nm unimodal sample at number concentrations of <*N*> = 6, 12, 24, 48, and different levels of noise. (**a**) 0, (**b**) 10^−4^, (**c**) 10^−3^, (**d**) 10^−2^, and (**e**) 10^−1^ noise levels.

**Figure 8:**
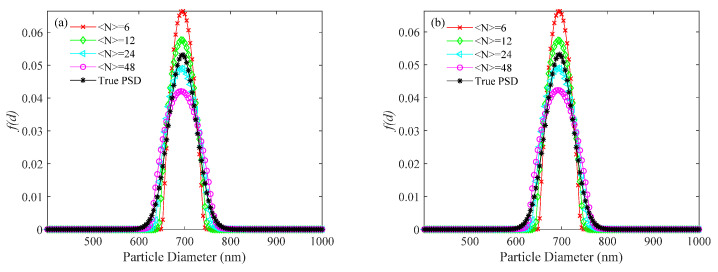

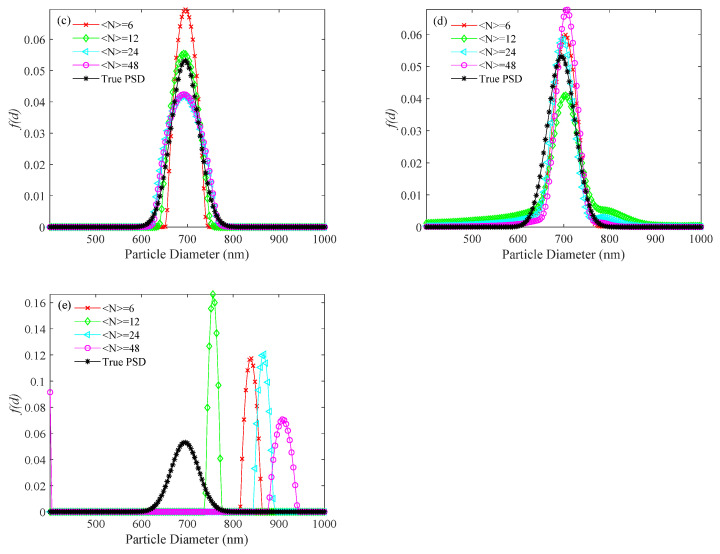
PSD recovered by the KFR method for a 696 nm unimodal sample at number concentrations of <*N*> = 6, 12, 24, 48, and different levels of noise. (**a**) 0, (**b**) 10^−4^, (**c**) 10^−3^, (**d**) 10^−2^, and (**e**) 10^−1^ noise levels.

**Figure 9:**
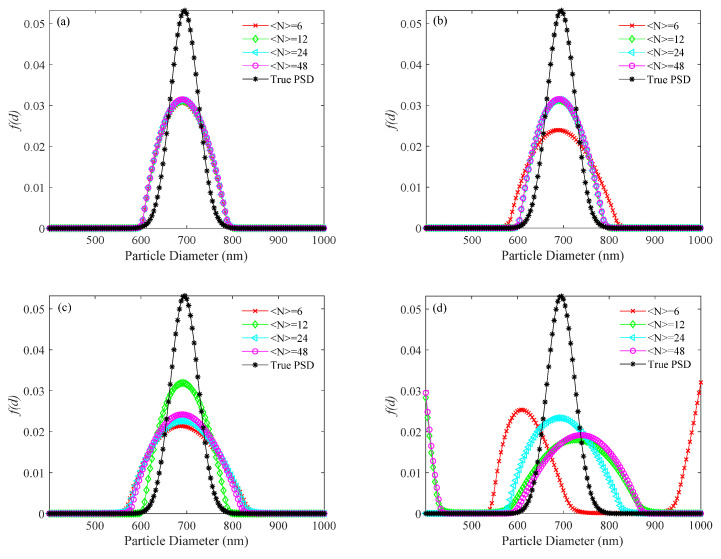

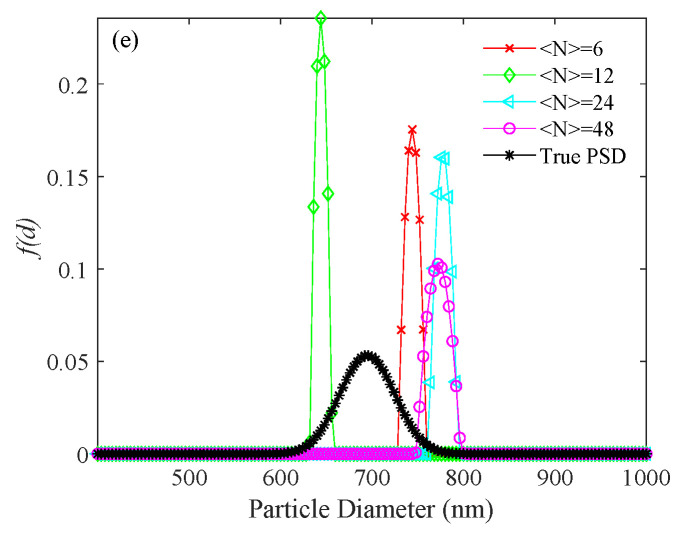
PSD recovered by the BR method for a 696 nm unimodal sample at number concentrations of <*N*> = 6, 12, 24, 48, and different levels of noise. (**a**) 0, (**b**) 10^−4^, (**c**) 10^−3^, (**d**) 10^−2^, and (**e**) 10^−1^ noise levels.

For the simulated 696 nm PSD, the PSDs recovered by the usual method, the KFR method and the BR method are shown in Figure 7, Figure 8 and Figure 9, respectively. Corresponding performance indices are shown in Table 4. At different noise levels, it is clear that false peaks obtained by the usual method exceed the upper limit of the PSD range at ultra-low concentrations. The appearance of false peaks causes the main peak to decrease and shift towards smaller particle sizes, and gradually move out of the lower limit of the PSD range as the concentration decreases. However, the KFR method gives better results. At 10^−3^ noise level, the relative error (*E_P_*_KFR_) and the recovery error (*V_E_*_KFR_) of the KFR method are as low as 0.006 and 0.01, respectively. When the noise level increased to 10^−1^, the performance indices deteriorated significantly. The BR method results are similar to those of the KFR method, but are slightly less affected by noise increase.

### 3.2. Experiment

**Figure 10:**
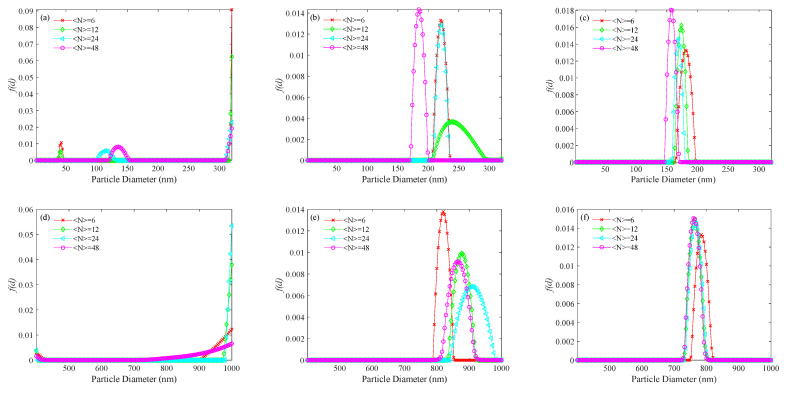
PSDs recovered for standard polystyrene latex spheres at number concentrations of <*N*> = 6, 12, 24, 48: 152 nm diameter sample; (**a**) the usual method, (**b**) the KFR method, and (**c**) the BR method; and the PSDs recovered for the 693 nm diameter sample; (**d**) the usual method, (**e**) the KFR method, and (**f**) the BR method.

Real data were obtained from an experimental setup including a solid-state laser with a wavelength of 532 nm (model MGL-III-532 nm–15 mW), a photon counter (model CH326, Hamamatsu Photonics), and a 512 channels digital correlator. The samples were dilute suspensions of latex spheres in distilled water. They were made from 152 ± 5 nm (Duke 3150A) and 693 ± 10 nm (GBW(E)120087) standard polystyrene latex spheres. The laser power was 15 mW, the scattering angle was 90°, the focal length of the incident lens was 175 mm, the receiving aperture of the detector was 400 μm, and the sample cell temperature was 298.15 K. The PSDs recovered for the 152 and 693 nm diameter spheres are shown in Figure 10 and the corresponding performance indices are shown in Table 5.

As is shown in Figure 10 and Table 5, the PSDs recovered by the KFR method and the BR method are better than those by the usual method. Strong false peaks in the PSDs recovered by the usual method did not appear in the PSDs recovered by the KFR method and BR method. At all concentrations, the relative errors of peak positions obtained by BR method are smaller than those obtained by KFR method, which means the BR method is better than the KFR method. For the 152 nm particles, the appearance of strong false peaks causes the main peak reflecting the true particle size to nearly disappear, and the peak position of the main peak shifts significantly to small sizes as the concentration decreases. For the 693 nm particles, the PSDs recovered by the usual method showed the main peak reflecting the true particle size in the PSD results has completely disappeared and only the false peaks remain. The experimental results from the KFR method are different from those with the simulated data. Although there are no more false peaks, the recovered peak positions are obviously shifted from the true positions. These PSDs with large peak position errors meaningless. For the BR method, the relative errors between the experimental PSDs and the recovered PSDs are closer to the simulations at high noise level. Compared with the recovered PSDs of simulations at low-noise conditions, the relative errors of the experimental PSDs using the BR method show a significant increase.

## 4. Discussion

**Figure 11:**
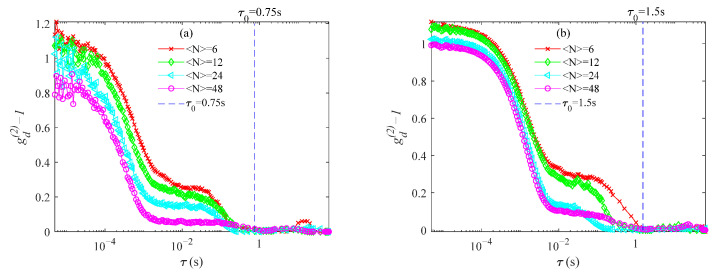
Measured ACFs of standard polystyrene latex particles for concentrations of <*N*> = 6, 12, 24, and 48: (**a**) 152 nm diameter and (**b**) 693 nm diameter.

**Figure 12:**
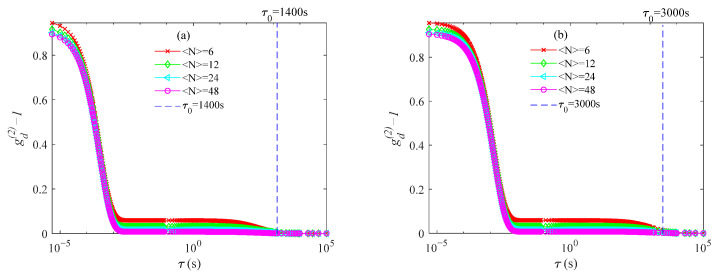
Simulated ACFs of particles for concentrations of <*N*> = 6, 12, 24, and 48: (**a**) 152 nm diameter and (**b**) 693 nm diameter.

The results from the simulated data show obvious false peaks in the PSDs obtained by the usual method at ultra-low concentrations and the peak positions exceed the upper limits of the PSD range. Since the normalized PSD satisfies $\sum_{i=1}^{M}f\left(d_{i}\right)=1$, large false peaks that exceed the upper limits of the PSD range are compensated by peaks at small sizes and accurate PSDs are not obtained. The KFR method, using a theoretical model that includes number fluctuations, should account for the number fluctuation term in the intensity ACF and produce reliable PSDs. This did occur with the simulated data, especially with low added noise. However, this was not the case with measured data. The KFR method did not produce the false peaks in the large particle size region, but it did not obtain peak positions that were consistent with or even close to those in the true PSDs. Figure 11 shows the measured intensity ACFs and it is clear that they contain more noise than the simulated ACFs, especially at long delay times. This noise is greater than normally found in ACFs from DLS due to the very low sample concentrations involved. The scattered light signal is weaker and the background noise is relatively larger due to the lower concentrations. Furthermore, at long delay times, the noise is more apparent in the intensity ACF. This region is essentially the ACF baseline at normal particle concentrations and it is usually dealt with using truncation or weighting methods [32,33]. However, this segment is located in the number fluctuation region of the ACFs at ultra-low concentration. If the data is noisy, it will be difficult for any model to recover the number fluctuation term successfully. Figure 12 shows the intensity ACFs for 152 and 693 nm particles with <*N*> = 6, 12, 24, and 48 calculated from Equation (8). In Figure 11 and Figure 12, the vertical dashed line marks the delay time τ_0_, when the ACF decays to 10^−3^ in the long delay time region. The τ_0_ of the measured and simulated ACFs are 0.75 s, 1.5 s for the 152 nm particles, and 1400 s, 3000 s for the 693 nm particles, respectively.

So, the measured ACFs appear to decay down to the level where the number fluctuation term would be significant. However, as can be seen from Figure 11, at that point the ACFs are dominated by noise and oscillations, so it is difficult to observe the number fluctuation term, much less estimate its level. Nijman et al. [34] also believed that noise was an issue and this was because the experiment time was too short. Our measurement time was 120 s, which was very short compared with the ideal measurement time of ~10^5^ s, assuming measuring an accurate ACF requires a measurement time of at least 10 correlation times.

Number fluctuation exists in scattering volume at any concentration. At normal concentrations ($\langle N\rangle <<{\langle N\rangle }^{2}$), number fluctuations may be ignored since there are a vast number of particles in the scattering volume. At ultra-low concentrations, the proportion of scattered light fluctuations caused by number fluctuations compared with those from Brownian motion increases as the concentration decreases and this is manifested in the ACF and cannot be ignored.

As seen in the simulations, the inversion results obtained by the KFR method are better than those of the BR method. Under the same conditions, the KFR method includes the full number fluctuation component that contains particle size information, while the BR method removes it. Since the number fluctuation component contains particle size information, although the information in this part is far less than it in the Brownian motion component, the BR method cannot give more accurate PSD than those of the KFR method. However, with increasing noise, compared with the benefits of including PSD information, the noise in number fluctuation component will gradually damage the accuracy of the PSDs. At the noise level of 10^−1^, inversion results by the KFR method get worse. Compared with the normal concentration, the noise in the measured data at low concentrations is more serious and appears more at the end section of the Brownian motion component in the intensity ACF, which aggravates the deterioration of the KFR method inversion results. The influence of noise also affects the reset of the ACF baseline. The accuracy of the inversion result of the BR method gets worse with increasing noise, which is also obvious in the inversion of the measured data. Although both methods cannot give ideal inversions for measured data, compared with KFR method, the BR method is only affected by noise on the truncation point of ACF, and is not affected by the negative effect of noise in the number fluctuation term. Therefore, in actual measurement, it appears that the BR method, as a theoretically unsatisfactory method, can give relatively better PSD results than a theoretically ideal method, the KFR method. Compared with the inversion results of simulated data, it can also be seen that the inversion results of measured data are closer to the inversion results of simulated data at 10^−1^ noise level, which reflects that the noise at ultra-low concentration is much higher than that at normal concentrations, and becomes an important inhibiting factor for accurate DLS measurements at ultra-low concentration.

## 5. Conclusions

In DLS measurements, particles entering and leaving the scattering volume give rise to number fluctuations and, therefore, scattered light intensity fluctuations which will manifest in the intensity ACF. When the number of particles in the scattering volume is large, the number fluctuation term in the ACF is usually many orders of magnitude less than the Brownian motion term and can be ignored. When 〈N〉<50, the contribution of number fluctuations to the ACF cannot be ignored. This is the higher limit of concentration used in our experiments. The key to this limitation is that the field ACF obtained from the measured intensity ACF cannot match with the usual kernel function matrix at ultra-low concentrations, which is based only on the diffusion of the Brownian particles. It is feasible to recover particle sizing information from ultra-low concentration DLS measurements if the number fluctuation term in the intensity ACF is accounted for.

This paper introduces two methods for doing this. The first is the KFR method, which specifically incorporates the number fluctuation term in the intensity ACF model used to reconstruct the kernel function matrix. The second is the BR method, which recognizes that the number fluctuation term has very low amplitude and decays very slowly so that it may be considered a baseline component of the ACF and resetting the baseline may account for it. The two methods were used to recover PSDs from simulated and measured ACF data. As is shown in results, ideal recovered PSDs can be obtained from the simulated data by including the number fluctuation term under the normal noise level, but this does not occur with experimental measured data, and the inversion results of experimental data are closer to those of simulated data with high level noise. The reason is that the measured intensity ACF contains more noise than the simulated ACF at ultra-low concentration. In particular, the baseline noise in the long delay time tail of the ACF overwhelms the number fluctuation term and makes it difficult to recover reliable PSD data. The effective removal of the number fluctuation term from the ACF by resetting the baseline, is also a feasible way to improve PSD recovery at ultra-low concentration. However, the increasing noise at ultra-low concentration leads to errors in determining the effective baseline. This significantly reduces the accuracy of the inversion results. To effectively use the PSD information in intensity ACF at ultra-low concentration, the first priority is to minimize the noise on the ACF. Then, we can further explore the methods proposed above.

## Figures and Tables

**Table 1:** The decrease in magnitude, $\Delta g^{\prime }$, of the number fluctuation term, $g^{\prime }\left(\tau \right)$, for 10 and 1000 nm particles in the Brownian motion decay time, $t_{c}$ and the delay time, $t_{d}$, at which the intensity ACFs, $g_{d}^{\left(2\right)}\left(\tau \right)$, decay to 0.001 for 10 and 1000 nm particles at 〈N〉 = 5, 15, and 50 particles in the scattering volume.

Particle Size (nm)	<*N*>	Δ*g*′	*t_c_* (s)	*t_d_* (s)
10	5	1.0 × 10^−8^	3.0 × 10^−5^	1.0 × 10^−4^
	15	3.3 × 10^−9^	3.0 × 10^−5^	5.0 × 10^−3^
	50	1.0 × 10^−9^	3.0 × 10^−5^	2.0 × 10^−3^
1000	5	1.6 × 10^−8^	4.0 × 10^−3^	1.0 × 10^−6^
	15	5.2 × 10^−9^	4.0 × 10^−3^	4.0 × 10^−5^
	50	1.6 × 10^−9^	4.0 × 10^−3^	2.0 × 10^−5^

**Table 2:** Parameters and properties of the simulated PSDs.

*P* (nm)	*μ*	*σ*	(*d*_min_, *d*_max_) (nm)
156	0.50	9.0	(2, 320)
696	0.17	5.0	(400, 1000)

**Table 3:** Performance parameter values for the recovery of the 156 nm PSD by using three methods.

*δ*	<*N*>	*P*_U_ (nm)	*P*_KFR_ (nm)	*P*_BR_ (nm)	*E*_*P*U_	*E*_*P*KFR_	*E*_*P*BR_	*V*_*E*U_	*V*_*E*KFR_	*V*_*E*BR_
	True	156	156	156	0	0	0	0	0	0
0	6	68/-	154	152	0.6	0.01	0.03	0.03	0.002	0.009
	12	88/-	154	152	0.4	0.01	0.03	0.03	0.003	0.009
	24	101/-	156	152	0.4	0	0.03	0.03	0.003	0.009
	48	115/-	156	152	0.3	0	0.03	0.02	0.005	0.009
1 × 10^−4^	6	55/-	154	152	0.6	0.01	0.03	0.03	0.006	0.009
	12	74/-	154	152	0.5	0.01	0.03	0.03	0.004	0.009
	24	91/-	154	152	0.4	0.01	0.03	0.03	0.004	0.009
	48	105/-	154	152	0.3	0.01	0.03	0.02	0.005	0.009
1 × 10^−3^	6	55/-	154	150	0.6	0.01	0.04	0.07	0.01	0.01
	12	76/-	154	150	0.5	0.01	0.04	0.06	0.006	0.01
	24	95/-	156	154	0.4	0	0.01	0.05	0.003	0.01
	48	103/-	156	154	0.3	0	0.01	0.05	0.005	0.01
1 × 10^−2^	6	61/-	173	141	0.6	0.1	0.1	0.03	0.02	0.03
	12	84/-	158	78	0.5	0.01	0.5	0.03	0.006	0.04
	24	120/-	180	122	0.2	0.2	0.2	0.03	0.03	0.04
	48	114/-	163	71	0.3	0.05	0.5	0.03	0.01	0.04
1 × 10^−1^	6	-/-	14/190	-/150/-	-	-	0.04	0.04	0.03	0.08
	12	-/99	4/190	-/178	0.4	-	0.1	0.04	0.04	0.07
	24	103/-	174/-	173	0.3	0.1	0.1	0.03	0.03	0.03
	48	108/-	155/-	161	0.3	0.006	0.03	0.03	0.007	0.01

**Table 4:** Performance indices for the recovery of the 696 nm PSD by using three methods.

*δ*	<*N*>	*P*_U_ (nm)	*P*_KFR_ (nm)	*P*_BR_ (nm)	*E*_*P*U_	*E*_*P*KFR_	*E*_*P*BR_	*V*_*E*U_	*V*_*E*KFR_	*V*_*E*BR_
	True	696	696	696	0	0	0	0	0	0
0	6	-	696	692	-	0	0.006	0.07	0.005	0.007
	12	-	692	692	-	0.006	0.006	0.05	0.003	0.006
	24	-	692	692	-	0.006	0.006	0.04	0.002	0.006
	48	420/-	692	692	0.4	0.006	0.006	0.03	0.003	0.006
1 × 10^−4^	6	-	696	688	-	0	0.01	0.07	0.005	0.009
	12	-	692	692	-	0.006	0.006	0.05	0.003	0.006
	24	-	692	692	-	0.006	0.006	0.04	0.002	0.006
	48	424/-	692	692	0.4	0.006	0.006	0.03	0.003	0.006
1 × 10^−3^	6	-	696	688	-	0	0.01	0.07	0.006	0.01
	12	-	692	692	-	0.006	0.006	0.05	0.002	0.006
	24	-	692	688	-	0.006	0.01	0.04	0.003	0.009
	48	424/-	692	692	0.4	0.006	0.006	0.03	0.003	0.009
1 × 10^−2^	6	-	704	608/-	-	0.01	0.1	0.07	0.004	0.02
	12	-	704	-/728	-	0.01	0.05	0.05	0.005	0.01
	24	-	696	688	-	0	0.01	0.04	0.003	0.003
	48	-	708	-/740	-	0.02	0.06	0.03	0.006	0.009
1 × 10^−1^	6	-	840	744	-	0.2	0.07	0.08	0.03	0.03
	12	408/-	756	644	0.4	0.09	0.08	0.07	0.03	0.04
	24	500/-	868	776	0.3	0.2	0.1	0.06	0.03	0.03
	48	-/-	-/908	772	-	0.3	0.1	0.06	0.02	0.03

**Table 5:** Performance indices for the recovery of 152 and 693 nm PSDs.

*P* (nm)	<*N*>	*P*_U_ (nm)	*P*_KFR_ (nm)	*P*_BR_ (nm)	*E*_PU_	*E*_*P*KFR_	*E*_*P*BR_
152	6	40/-	220	180	0.7	0.4	0.2
	12	42/-	239	173	0.7	0.6	0.1
	24	114/-	220	169	0.3	0.4	0.1
	48	135/-	184	156	0.1	0.2	0.03
693	6	-	820	784	-	0.2	0.1
	12	-	876	764	-	0.3	0.1
	24	-	908	768	-	0.3	0.1
	48	-	864	760	-	0.3	0.1

## Data Availability

Not applicable.
